# On the Limited Potential of Azorean Fleshy Fruits for Oceanic Dispersal

**DOI:** 10.1371/journal.pone.0138882

**Published:** 2015-10-14

**Authors:** Carolina Franco Esteves, José Miguel Costa, Pablo Vargas, Helena Freitas, Ruben Huttel Heleno

**Affiliations:** 1 Centre for Functional Ecology, Department of Life Sciences, University of Coimbra, Coimbra, Portugal; 2 Real Jardín Botánico de Madrid (CSIC-RJB), Madrid, Spain; Institute of Botany, CHINA

## Abstract

How plants arrived to originally sterile oceanic islands has puzzled naturalists for centuries. Dispersal syndromes (i.e., diaspore traits that promote dispersal by long-distance dispersal vectors), are generally considered to play a determinant role in assisting island colonization. However, the association between diaspore traits and the potential vectors by which diaspores are dispersed is not always obvious. Fleshy fruits, in particular, are considered to have evolved to promote the internal dispersal of seeds by frugivores (endozoochory), however some fleshy fruits can also float in saltwater, and thus be potentially transported by oceanic current (thalassochory). We performed saltwater floatation and viability experiments with fruits of the 14 European fleshy-fruited species that naturally colonized the Azores archipelago (North Atlantic Ocean). We show that only *Corema album* (a berry) and *Juniperus oxycedrus* (a fleshy cone) floated for as long as 60 days, the estimated minimum time needed to reach the Azores by oceanic currents. Regardless the floatation potential, exposure to saltwater largely reduced the viability of most seeds of the 14 species (46% of viability decline within 15 days and 77% within 60 days of immersion), including those of *Corema album* (61%) and *Juniperus oxycedrus* (83%). Floatability and viability trials suggest that while some fleshy-fruited species might have arrived to the Azores by oceanic currents, such would have required extreme meteorological events that could largely reduce the duration of the trip. Thus, the alternative hypothesis that fleshy-fruited species were mostly dependent on animal dispersers (endozoochory) to colonize these remote islands is reinforced.

## Introduction

Seed dispersal, and especially long-distance dispersal (LDD), is a key process that allows the colonization of new habitats and the maintenance of spatial vegetation dynamics [1–3]. LDD traits are particularly important for the colonization of isolated oceanic islands, i.e. those that never had a land bridge to a continent and thus received all their biota by LDD [4,5]. Recent developments (e.g. geological sciences and molecular tools) in phylogeography have revived an interest on the drivers of biogeographic patterns and on the consequences of LDD to and from islands [4,6]. Early naturalists were well aware of the importance of seed dispersal for biogeography and several classical studies tried to infer the mechanisms responsible for the assembling of insular floras by analysing the morphological traits of the diaspores (the propagative part of the plant) [7–10], and specifically their capacity to float and survive saltwater immersion [9,10]. However, studying the specific vectors that drive LDD colonization events based on empirical data is inherently challenging [11–13], particularly due to the predicted importance of non-standard dispersal processes [14].

Many plant species acquired fruits with specialized morphological traits such as wings or hooks that are considered to enhance the probability of dispersal by specific vectors, e.g. wind and animals, respectively. These traits can be grouped into dispersal syndromes based on the vectors to which they are especially well-adapted [15]. Although a variety of dispersal syndromes have been identified, only four can give a relevant contribution to LDD on a biogeographic context of island colonization: endozoochory (dispersal by ingestion and ejection of seeds by animals), epizoochory (external transport of diaspores by animals), anemochory (dispersal by wind), and thalassochory (dispersal by saltwater—i.e. oceanic currents) [3,16,17]. While the relevance of these traits for promoting actual colonization has been historically assumed, recent studies showed that the actual dispersal vectors by which seeds are transported cannot be directly inferred from diaspore morphology, since non-standard vectors can also have an important and largely unpredictable effect on colonization [14,16]. Therefore, even if standard dispersal vectors are responsible for the dispersal of most seeds, the study of non-standard vectors, such as the transport of fleshy fruits by oceanic currents or the internal transport of winged seeds after accidental ingestion, can become pivotal in explaining many observed colonization patterns, and particularly the long-distance colonization of remote islands [14,16]. The relevance of non-standard dispersal mechanisms will be particularly important when the alternative dispersal vector can largely affect the dispersal distance relatively to seeds dispersed by the standard dispersal mechanism [14]. For example, when seeds with no specific anchorage structures adhere to the feathers or fur of animals [18]. In this respect oceanic currents can be particularly relevant given their potential to transport diaspores over very large distances. Such an effect has been detected in the colonization of the island of Surtsey with many plants probably being transported by sea currents for c. 30 km, many of which lacking specific adaptations to this type of dispersal [14,19]. More striking examples of the likely importance of oceanic long distance dispersal include the movement of seeds between Tasmania and New Zealand (c. 1500 km), Galápagos and South America (c. 1000 Km) and the Azores and Europe (c. 1400 km) [16,20,21].

The morphological traits associated with anemochory and epizoochory can often be readily recognized from an external examination of the diaspore features, due to the presence of plumes, wings or pappus that promote the prolonged suspension on atmospheric air currents, and barbs, hooks, spines or viscid mucilage to enable the external adhesion of seeds to the body of animals [3,17,22]. Adaptations to stimulate fruit ingestion by animals typically consist of nutritive tissues surrounding the diaspores, largely corresponding to fleshy fruits [17]. Finally, specializations to oceanic currents involve special low-density tissues surrounding the diaspores that promote floatation in saltwater [9,10] and survival of spermatophyte embryos [23]. Thus, the interpretation on whether the tissues surrounding the diaspores are an adaptation to attract frugivores (endozoochort) or a buoyancy structure (thalassochory), depends on the physical and chemical characteristics of the tissues, such as density, caloric content, water content, secondary compounds, and palatability [3]. For this reason, the association of diaspore traits and these two dispersal syndromes is not always obvious [14,16,21,24]. Furthermore, while anemochory and epizoochory are not likely to alter the germination potential of seeds, the seeds transported either by saltwater or in the animal guts have to endure highly abrasive physical and chemical conditions and thus seed viability can be affected [2,10,23]. Several studies evaluate diaspore buoyancy and viability after immersion in saltwater, particularly of small and dry seeds [9,10,25]; nevertheless, research on seed viability after animal ingestion is far ahead than that of seed survival after saltwater exposure [3,26].

Although endozoochory has been classically suggested as a key mechanism for the colonization of islands by plants [27], there is also solid evidence that thalassochory might have played a pivotal role in the colonization of several archipelagos [9,21,28,29] and particularly the Azores [16]. Nevertheless, the capacity of fleshy fruits to be dispersed by oceanic currents (resulting from their floatability and viability after prolonged exposure to saltwater) cannot be evaluated without empirical experimentation [3,7,10,16].

Here we empirically tested the potential of native fleshy-fruited plants from the Azorean for being dispersed by oceanic currents based on the recent finding that species with thalassochorous traits are overrepresented in this archipelago when compared to continental (source) flora [16]. Specifically, we tested the floatability and viability of the diaspores of the 14 fleshy-fruited species representing 14 genera native to the Azores after moderate and prolonged immersion in saltwater. In order to cross-validate the results of the viability tests, seed viability was further tested by performing glasshouse germination trials with seeds submitted to each experimental treatment.

## Methods

**Fig 1:**
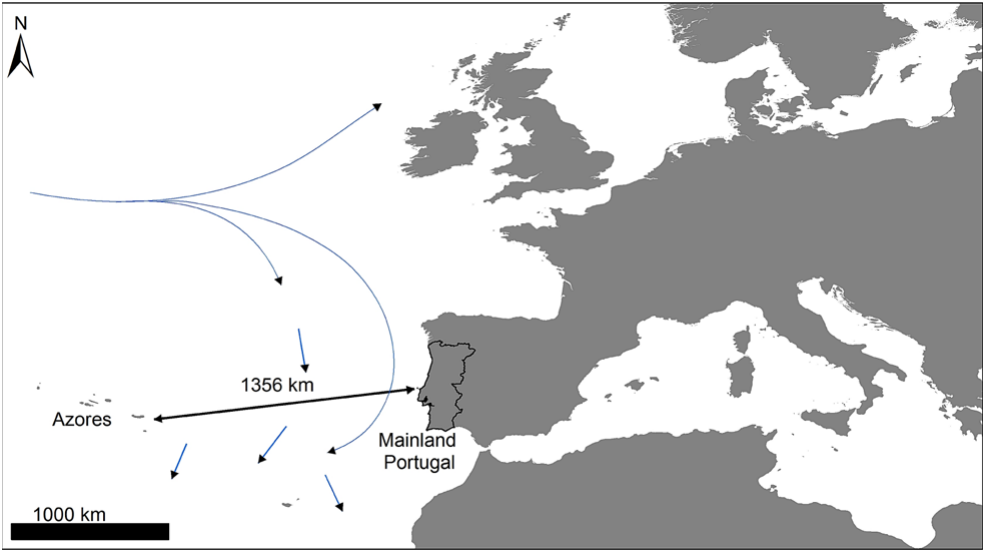
Global position of the archipelago of the Azores in relation to Europe. Map of the Azores, Europe, and the main oceanic current systems in the North Atlantic (36–39°N, 25–31°W).

The flora of the Azores, a volcanic archipelago in the North Atlantic Ocean (Fig 1) mostly colonized from the European continental flora [30–32], offers an ideal experimental framework to test hypothesis on the relationship between diaspore traits and their capacity for LDD. The nine islands of the Azores are characterized by a mild oceanic climate with high relative humidity [33]. The current Azorean flora is formed by 148 native species, of which approximately one-third are endemic, and c. 800 species introduced by man since the islands were first discovered [34,35].

In order to evaluate the capacity of fleshy fruits to be dispersed by oceanic currents from continental Europe to the Azores, we experimentally tested the floatability and viability of fruits and seeds, respectively of all fleshy-fruited plant species shared by both floras after moderate and prolonged immersion in saltwater (S1 Table). Because the goal was to evaluate the dispersal potential of the ancestral plant lineages that colonized the Azores, the fruits for the experiments were collected along the coast of mainland Portugal (S1 Table). When the species currently found in the Azores was not present on the mainland (i.e. endemic Azorean species), we used closely-related species on the mainland based on available phylogenetic reconstructions [36,37]. This approach intended to test the dispersal performance of the most likely continental coloniser. In particular, sister-group relationships were considered for 10 of the 14 fleshy-fruited species endemic to Azores as a result of analysing previous phylogenetic results (Table 1). Due to difficulties in finding fruiting populations of *Vaccinium myrtillus*, *Daphne laureola*, and *Juniperus navicularis*, floatability and viability tests for these genera were conducted with fruits of *V*. *cylindraceum*, *D*. *gnidium*, and *J*. *oxycedrus*, respectively (S1 Table) [36]. At the moment it is not absolutely clear if the closest relative of *Smilax azorica* is European or American [38,39], nevertheless, we decided to include *S*. *aspera* in the trials due to its similar fruit morphology with *S*. *azorica*. Fruits for the trials were collected during autumn and winter 2013/2014 in natural populations and experiments were conducted immediately after collection (no specific permissions were required for these activities because the collection was not carried out on privately owned land or National Parks).

**Table 1:** Proportion of floating and viable (Tetrazolium test) seeds for 14 European fleshy-fruited species after 60 days of immersion in saltwater, and the inferred potential for oceanic dispersal. Taxonomy of Azorean species followed [34], European taxonomy followed the Flora Europaea of selected plant families [43].

Origin	Family	Azorean species	Species in the trials	Floating fruits	Viable seeds	Thalassochorous potential
Endemics	*Rhamnaceae*	*Frangula azorica*	*Frangula alnus*	0%	71%	None
	*Araliaceae*	*Hedera azorica*	*Hedera hibernica*	0%	0%	None
	*Aquifoliaceae*	*Ilex perado sub*. *azorica*	*Ilex aquifolium*	0%	5%	None
	*Cupressaceae*	*Juniperus brevifolia*	*Juniperus oxycedrus*	0.07%	17%	Residual
	*Lauraceae*	*Laurus azorica*	*Laurus nobilis*	0%	85%	None
	*Rosaceae*	*Prunus azorica*	*Prunus lusitanica*	0%	0%	None
	*Rosaceae*	*Rubus hochstetterorum*	*Rubus ulmifolius*	0%	41%	None
	*Smilacaceae*	*Smilax azorica*	*Smilax aspera*	0%	8%	None
	*Ericaceae*	*Vaccinium cylindraceum*	*Vaccinium cylindraceum*	0%	0%	None
	*Adoxaceae*	*Viburnum treleasei*	*Viburnum tinus*	0%	27%	None
Non-endemic natives	*Ericaceae*	*Corema album*	*Corema album*	0.36%	39%	Residual
	*Thymelaeaceae*	*Daphne laureola*	*Daphne gnidium*	0%	0%	None
	*Myricaceae*	*Morella faya*	*Morella faya*	0%	13%	None
	*Taxaceae*	*Taxus baccata*	*Taxus baccata*	0%	24%	None

### Floatability

We tested diaspore floatability in saltwater by placing 50 fruits of each species in 20 x 20 x 10 cm containers with sea water (taken from the Atlantic Ocean) and recording the number of floating fruits each day for 60 days (S1 Fig). Water containers were shaken for variable lengths of time (between 5 and 180 minutes) and with variable intensities (between 30 to 50 rpm) every day, on a GFL® Orbital Shaker 3005. These variable conditions intended to simulate the natural variability of physical stress of the sea surface. It is important to note that our experiments only tested for the potential of diaspores to endure the trip from Europe to the Azores, however if they actually complete the crossing is contingent on other external factors, such predation at sea by marine wildlife. Water was carefully replaced weekly to maintain oxygenation and avoiding eutrophication. The number of floating diaspores was recorded every day and the oceanic dispersal potential of diaspores (i.e. buoyancy) was calculated as the proportion of diaspores still floating after moderate (15 days) and prolonged (60 days) immersion. The prolonged immersion period was calculated as the minimum time needed for a floating seed to reach the Azores based on the maximum estimated sea current velocity (30 cm.s^-1^, S2 Table) [15], while 15 days would allow inter-island colonization given the mean current velocity and maximum inter-island distance (at c. 20 cm.s^-1^, a seed could travel approximately 260 km within 15 days, which encompasses most inter-island distances within the archipelago)[15]. At the end of the experiment, the number of floating and sunk diaspores was counted and their viability tested. For each species, differences in the viability of diaspores under both treatments (15 and 60 days) were contrasted with the viability of control seeds using likelihood ratio tests (G tests, α = 0.05) performed in R [40].

### Viability

We tested the viability of seeds from diaspores under three treatments: 1) non-immersed fruits (control); 2) fruits immersed for 15 days, and 3) fruits immersed for 60 days in saltwater (both floating and sunk in the last two treatments). Seed viability was evaluated by exposing 20–24 seeds of each treatment to a staining test by 2, 3, 5 triphenyl-tetrazolium chloride solution (TTC) [41], see details in [42]. The optimal concentration of TTC used for each species was experimentally selected *a priori* by selecting from three concentrations (1%, 0.5% and 0.1%) the one with the best staining pattern. The embryos were carefully extracted from all seeds and hydrated for 24h in distilled water before being subjected to the TTC (S2 Fig). In order to minimize the risk of damaging the embryos during the extraction, extra seeds of all species were collected in the field and embryo extraction of each species was practiced previous to the start of the viability experiments. Most fruits contained multiple seeds, thus only undamaged embryos were used for the viability tests. Bare embryos were immersed for 24h in TTC at room temperature and in darkness [41,42] after which they were observed with a binocular microscope and sorted into two categories according to their staining pattern: 1) Potentially viable (total or partial staining of meristematic regions of the shoot and root apices); and 2) Non-viable (not stained or stained only on non-meristematic tissues). The remaining 30 seeds per treatment/species were soaked in distilled water at room temperature for 24 hours and sown on a glasshouse for germination trials. Seeds were sown individually on germination trays with 36 cm^3^ wells using standard substrate (50% enriched soil and 50% sand), and individually labelled (S3 Fig). Germination trays were inspected daily during one year for newly emerged seedlings and watered whenever necessary.

## Results

**Fig 2:**
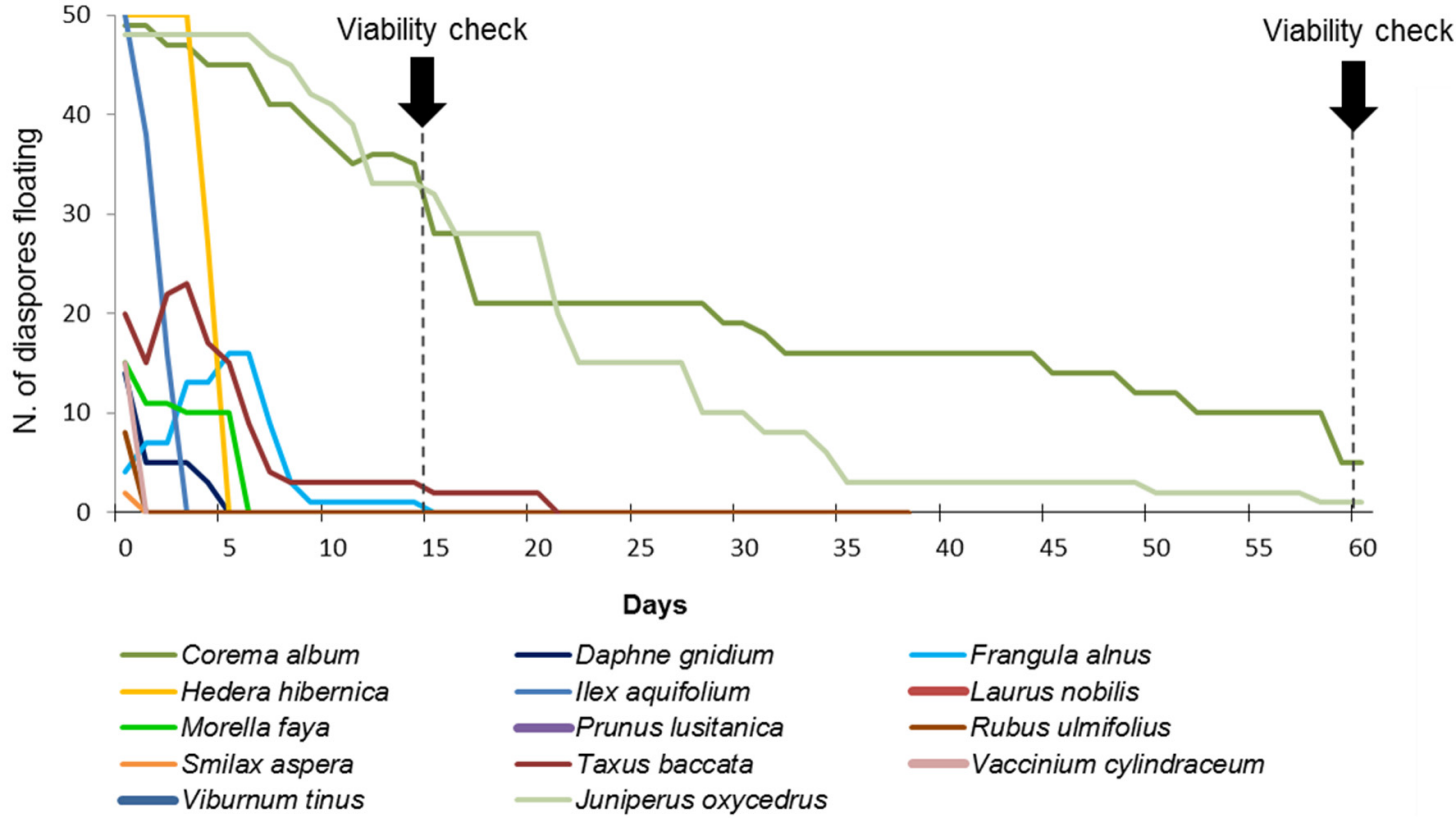
Number of fruits remained floating during prolonged immersion in saltwater. Floatation potential of Azorean fleshy fruits. Each line represents the fate (floating/sunk) of 50 fruits from each of the 14 fleshy-fruited species that naturally colonized the Azores.

**Fig 3:**
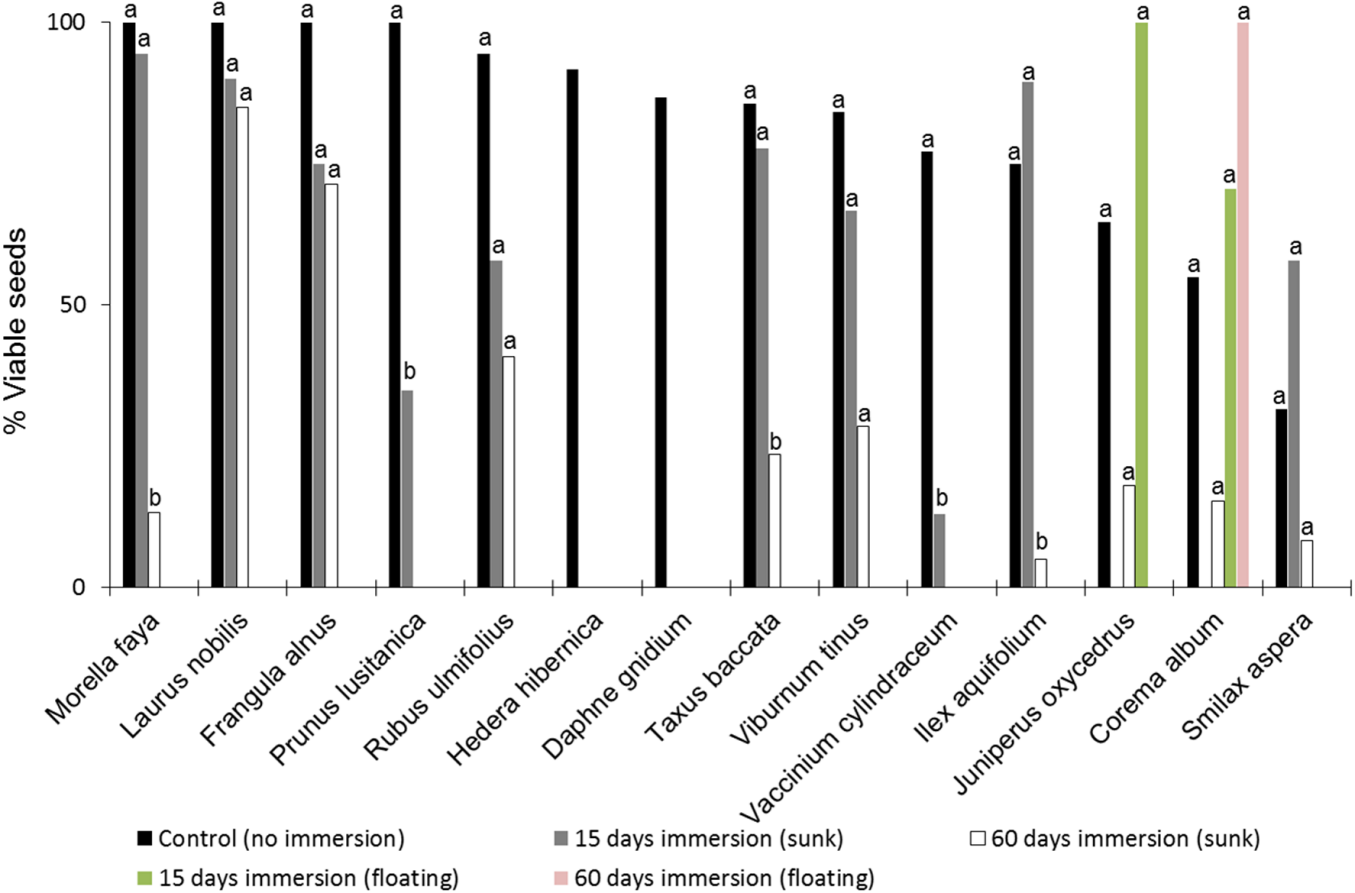
Percentage of viable embryos according to the tetrazolium (TTC) staining test. Embryos were tested under three treatments: control (no immersion), after moderate (15 days) and prolonged (60 days) immersion in saltwater. Different letters indicate significant differences between treatments and the control (G tests, α = 0.05), similar letters reflect no significant differences.

Overall, 2,100 diaspores from the 14 fleshy-fruited species native to the Azores were tested for floatability and viability (Table 1). At least some fruits of all species floated initially, but the majority of the fruits sank during the first five days of the experiment (Fig 2). Only 62 fruits (4.4%) from three species floated for longer than 15 days and only six fruits (0.4%) were still floating after 60 days: five fruits of *Corema album* (all with viable seeds) and one fleshy cone of *Juniperus oxycedrus* (not viable). Eight other species had at least one viable diaspores after 60 days immersion in saltwater, however none of these species floated for more than 20 days (Figs 2 and 3).

The proportion of viable seeds was reduced with prolonged exposure to saltwater for all species (Fig 3). All embryos of *D*. *gnidium* and *Hedera hibernica* lost their viability after moderate exposure to saltwater (Fig 3; S3 Table). Two species suffered a significant loss of viability between control and moderate exposure to saltwater (*Prunus lusitanica*, G = 4.26, d.f. = 1, p = 0.039; and *V*. *cylindraceum*, G = 8.22, d.f. = 1, p = 0.004), with no viable embryos after prolonged immersion (Fig 3; S3 Table). Three species only showed significant loss of viability after prolonged immersion (*Ilex aquifolium*, G = 11.16, d.f. = 1, p < 0.001; *Morella faya*, G = 8.03, d.f. = 1, p = 0.005; *Taxus baccata*, G = 4.57, d.f. = 1, p = 0.032) (Fig 3; S3 Table). Finally, seven species did not show a significant decrease in viability with immersion treatment (*C*. *album*, *Frangula alnus*, *J*. *oxycedrus*, *Laurus nobilis*, *Rubus ulmifolius*, *Smilax aspera*, and *Viburnum tinus*, for all these species G < 3.37, d.f. = 1, p > 0.066) (Fig 3; S3 Table).

Overall, 229 seeds of nine species germinated in glasshouse trials (S3 Table). Half of these seeds (51.1%, n = 117) were from non-immersed (control) fruits, 34.5% (n = 79) were from fruits immersed for 15 days, and the remaining 14.4% (n = 33) were from fruits immersed (sunk) for 60 days. None of the seeds retrieved from fruits still floating after 60 days germinated until the end of the experiment (S3 Table).

## Discussion

The performance of 12 of the 14 Azorean fleshy-fruited species suggests that thalassochory is an highly unlikely mechanism for the arrival of colonizing propagules to the Azores as their diaspores either do not float or cannot remain viable after prolonged immersion in saltwater. According to our results, only *C*. *album* and *J*. *oxycedrus* could have potentially colonized the Azores via fruits washed away from the shores of Europe and drifting on ocean currents. These species have, respectively, spongious and fibrous tissues around the seeds that facilitate diaspore floatation. However, even for these species, thalassochory remains an unlikely dispersal vector due to the reduced floatation capacity and low viability of immersed diaspores. The relatively large proportion of floating diaspores during the first days of the experiment probably corresponds to fruits with undeveloped embryonic tissues that rapidly absorbed water and sunk in a few days.

Seeds from all nine species germinated in the glasshouse and were thus viable, although we cannot conclude that seeds that did not germinate within 12 months were not viable, as they could remain dormant due to internal (genetic) or external (environmental) factors [44]. Moreover, the control germination results for *F*. *alnus* and *M*. *faya* are negatively biased due to overwatering shortly after sowing that likely constrained germination in these species through oxygen deprivation. Therefore, the results of the viability (TTC) tests offer a more reliable method to compare the effect of each treatment as they are not subjected to biological (e.g. seed dormancy) and environmental (e.g. temperature) constraints that influence seed germination [25,42].

The main oceanic drift system between continental Portugal and the Azores is a broad, slow and generally southward-flowing current known as the Portugal Current [45] which is part of a generally faster North Atlantic Currents System [46]. Considering the current velocity and direction of the Portugal Current (2.9 cm.s-1) [45], thalassochorous dispersal between Europe and the Azores would require at least 18 months. While it is possible that North Atlantic sea surface currents flowed at slightly higher velocities during past glaciation cycles, it is estimated that past velocities, and thus travel time, were within the same order of magnitude to present-day currents [46]. The colonization of the Azores might have been facilitated by former islands acting as stepping stones for biota, particularly the currently immersed Great Meteor archipelago located 800 km south of the Azores [32]. However, even considering this possibility, the journey would still require dispersal over very large stretches of sea (>800 km) which would be unlikely considering the reduced thalassochorous potential of the Azorean fleshy fruits. Anemochory is not a plausible mechanism for the transport of fleshy fruits from Europe to the Azores, given the considerable weight of the fleshy propagules and the eastwards direction of the prevailing winds. Azorean birds are known to consume and disperse the seeds of at least 43 species [47,48], and given the present results, this might have been a critical mechanism for the colonization of the Azores by fleshy fruits. The role of birds in seed dispersal is not restricted to frugivorous birds, but includes large omnivorous birds which tend to have longer gut retention times [12,49] and also seeds that escape predation by typical granivorous birds [50]. Alternatively, it is also possible that seeds of some fleshy fruits can externally attach to bird’s feathers during migration [18]. Some European birds species, which are potential dispersers of European propagules, are regularly observed in the Azores when stranded from their original migration routes [51,52]. However, it is puzzling that American bird species, representing 67% of the vagrant bird species recorded in the Azores have not resulted in an appreciable colonization event from New World plants. In particular, there are two species in the genus *Corema*, one facing Azores from the Western Europe (*C*. *album*) and the other from North-eastern America (*C*. *conradii*), but colonization has apparently only been successful from Europe. Similarly, the Gulf Stream could potentially transport thalassochorous diaspores to the Azores, but few representatives from North America (e.g. *Calystegia soldanella* and *Calystegia sepium* ssp. *americana*) are found in the Azores and these were probably introduced by man [31].

Other possible vectors might have included extreme meteorological events that were capable of transporting seeds very quickly [53] or driftwood, i.e. when whole trees or groups of trees are dragged to the sea still bearing some fruits that might remain adhered to the floating tree avoiding total or prolonged immersion [54,55].

Long distance dispersal events are intrinsically difficult to study [3]. Recent advances on molecular tools and facilitated access to high quality datasets are encouraging a revived interest on island colonization by plants [3,4,16]. A central difficulty is to distinguish between potential dispersal mechanisms and the real vectors responsible for the transport of each colonizing diaspore [14]. Although probabilistic approaches incorporating rapidly growing datasets can shed light on that relationship, we argue that classic empirical tests on the potential of seeds to endure the dispersal by oceanic currents and via animal ingestion are paramount and are not outdated.

## Conclusions

The present study shows that experimentation is needed to fully understand the dispersal potential of diaspores, which cannot be readily inferred from direct observation. The fruits of *C*. *album* offer a good example of the value of experimental clarification of dispersal syndromes as their fruits may have benefited from an initial dispersal assisted by birds (Europe to the Azores), but also the dispersal by oceanic currents between Azorean islands or even a combination between both vectors.

Considering the present surface currents system in the North Atlantic, and in the absence of extreme meteorological events, it is highly unlikely that thalassochory has been a relevant mechanism during the colonization of the Azores from the European continent by fleshy-fruited species. *Corema album* and *J*. *oxycedrus* seem the only species with some potential for dispersal via oceanic currents, particularly within the islands of the archipelago. Therefore, the alternative hypothesis that most fleshy fruits are highly dependent from endozoochory, and particularly ornithochory for long distance dispersal is herein reinforced as “*the less impossible of all the impossible speculations*” [31].

## Supporting Information

S1 Fig Trials of fruit floatability. We applied plastic containers filled with sea water and shaken on a GFL® Orbital Shaker 3005 in order to simulate oceanic physical stress conditions.
(TIF)

S2 Fig Seed viability test using 2, 3, 5 triphenyl-tetrazolium chloride solution (TTC). Tests for three treatments [41,42]: non-immersed fruits (control), after moderate (15 days) and prolonged (60 days) immersion in saltwater: **A.** embryos of *Prunus lusitanica* extracted and submerged in TTC at room temperature and darkness, **B.** and **C.** potentially viable embryos (total or partial staining of meristematic regions of the shoot and root apices, respectively), **D.** non-viable embryo (not stained or stained only on non-meristematic tissues).
(TIF)

S3 Fig General aspect of the germination tray. This image shows Laurus nobilis seeds submitted to the three treatments (control, moderate and prolonged immersion in saltwater) after six months on the glasshouse.
(TIF)

S1 Table Characterization of the species used in the viability and germination trials. Taxonomy of Azorean species followed [34], European taxonomy followed the Flora Europaea of selected plant families [43].
(DOCX)

S2 Table Different estimated speeds for the superficial oceanic currents (cm.s^-1^) between continental Portugal and the Azores. (DOCX)

S3 Table Results of viability tests (with Tetrazolium) and germination trials for 14 European fleshy-fruited species after 15 and 60 days of immersion in saltwater. Taxonomy of Azorean species followed [34], European taxonomy followed the Flora Europaea of selected plant families [43]. The proportion of viable and germinated seeds between the two treatments and the control have been tested with G-tests (d.f. = 1, α = 0.05) and statistically significant differences highlighted in bold.
(DOCX)
