# Supplementary figures and images for: On the Limited Potential of Azorean Fleshy Fruits for Oceanic Dispersal
Source: PLoS One. 2015 Oct 14;10(10):e0138882. doi: 10.1371/journal.pone.0138882 (PMC4605496; doi:10.1371/journal.pone.0138882)

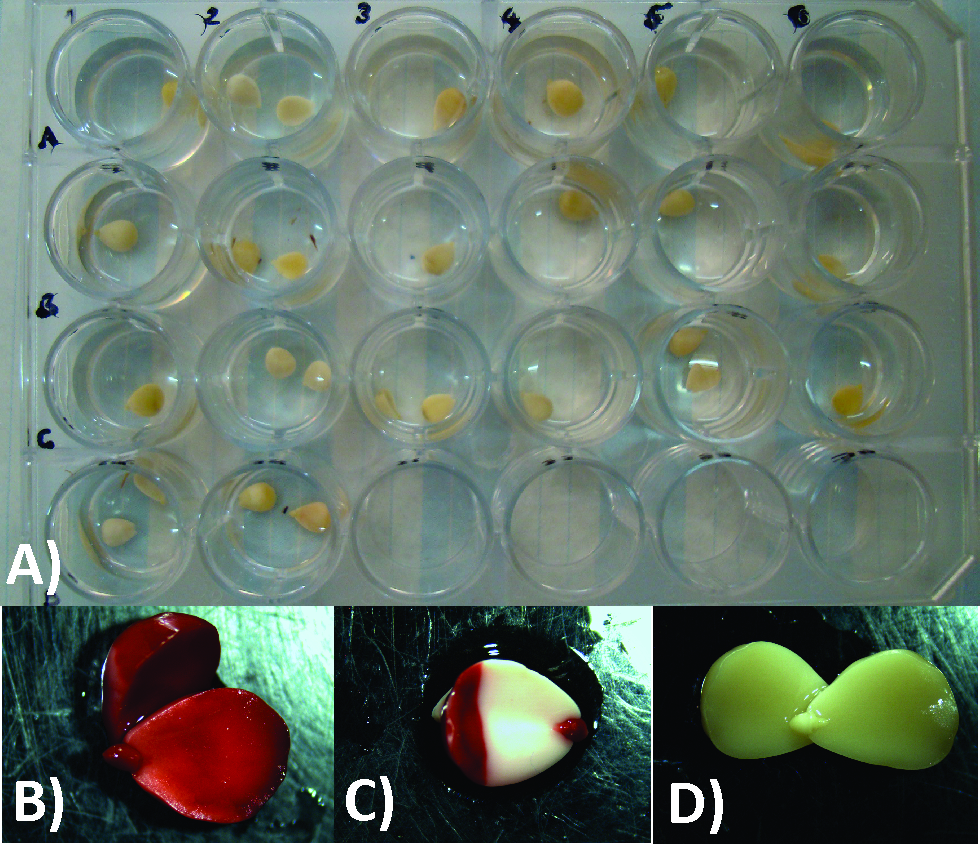

Supplement: S2 Fig — Tests for three treatments [41,42]: non-immersed fruits (control), after moderate (15 days) and prolonged (60 days) immersion in saltwater: A. embryos of Prunus lusitanica extracted and submerged in TTC at room temperature and darkness, B. and C. potentially viable embryos (total or partial staining of meristematic regions of the shoot and root apices, respectively), D. non-viable embryo (not stained or stained only on non-meristematic tissues). (TIF) [file pone.0138882.s002.tif]

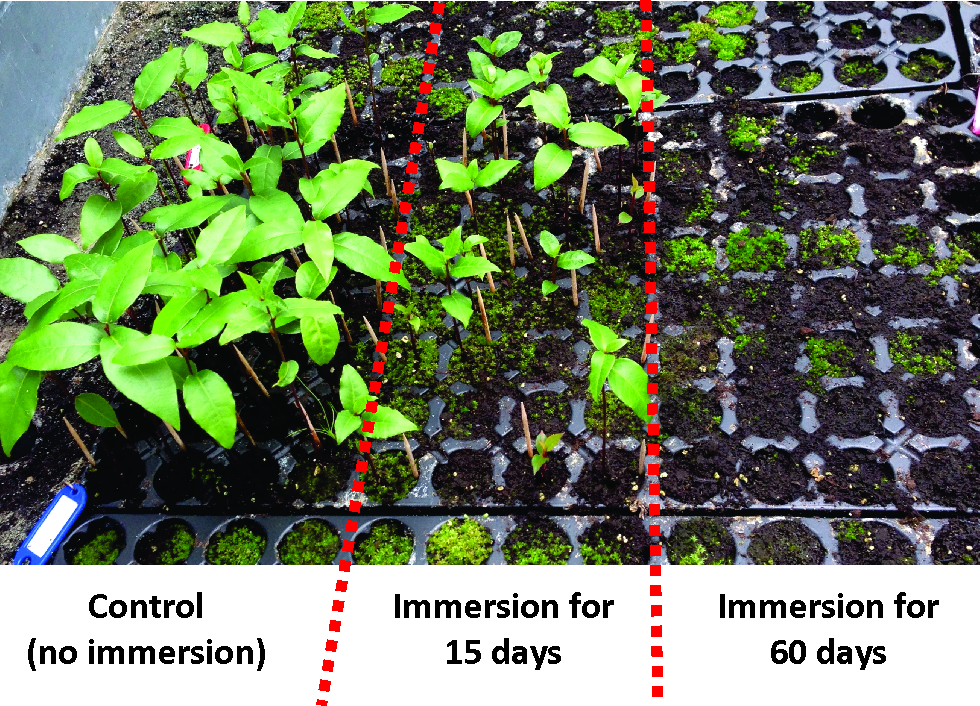

Supplement: S3 Fig — This image shows Laurus nobilis seeds submitted to the three treatments (control, moderate and prolonged immersion in saltwater) after six months on the glasshouse. (TIF) [file pone.0138882.s003.tif]
