# Supplementary material for: On the Limited Potential of Azorean Fleshy Fruits for Oceanic Dispersal
Source: PLoS One. 2015 Oct 14;10(10):e0138882. doi: 10.1371/journal.pone.0138882 (PMC4605496; doi:10.1371/journal.pone.0138882)
Supplement: S1 Table — Taxonomy of Azorean species followed [34], European taxonomy followed the Flora Europaea of selected plant families [43]. (DOCX) [file pone.0138882.s004.docx]

**S1 Table**

| **Azorean species** | **Status** | **Plant species used in the trials** | **Reference for phylogeny** | **Family** | **Collection date** | **Collection site (UTM coordinates)** |
| --- | --- | --- | --- | --- | --- | --- |
| *Corema album* (L.) D. Don subsp. *azoricum* P. Silva | Non-endemic | *Corema album* (L.) D. Don | - | Ericaceae | 2-Sep-2013 | São Pedro de Moel (39°45'07.2"N; 9°01'39.6"W) |
| *Daphne laureola* L. | Non-endemic | *Daphne gnidium* L. *(fruits of D. laureola could not be found)* | [1] | Thymelaeaceae | 28-Oct-2013 | Portela da Cerdeira (40°15'18.2''N; 7°56'59.3''W) |
| *Frangula azorica* V. Grubov | Endemic | *Frangula alnus* Mill. |  | Rhamnaceae | 4-Sep-2013 | Paúl de Madriz (40°07'47.4"N; 8°37'52.4"W) |
| *Hedera azorica* Carrière | Endemic | *Hedera hibernica* (G.Kirchn.) Kny | [2] | Araliaceae | 16-Fev-2014 | Dreia (40°14'55.4''N; 7°56'53.5''W) |
| *Ilex perado* Aiton subsp. *azorica* (Loes.) Tutin | Endemic | *Ilex aquifolium* L. | [3] | Aquifoliaceae | 8-Nov-2013 | Serra do Buçaco (40°22'11.2''N; 8°21'54.8''W) |
| *Juniperus brevifolia* (Seub.) Antoine | Endemic | *Juniperus oxycedrus* L. *(cones of J. navicularis could not be found)* | [4] | Cupressaceae | 15-Oct-2013 | Nozelos (41°45'19.3"N; 7°17'26.7"W) |
| *Laurus azorica* (Seub.) Franco | Endemic | *Laurus nobilis* L. | [5] | Lauraceae | 27-Oct-2013 | Vila Cova do Alva (40°16'58.2''N; 7°56'51.5''W) |
| *Morella faya* (Aiton) Wilbur | Non-endemic | *Morella faya* (Aiton) Wilbur | - | Myricaceae | 10-Sep-2013 | Praia de Mira (40°26'40.9''N; 8°47'23.1''W) |
| *Prunus azorica* (Hort. ex Mouillef.) Rivas Mart., Lousã, Fern. Prieto, E.Dias, J.C.Costa & C.Aguiar | Endemic | *Prunus lusitanica* L. | [6] | Rosaceae | 23-Sep-2013 | Mata da Margaraça (40°12'56.5''N; 7°54'43.9''W) |
| *Rubus hochstetterorum* Seub. | Endemic | *Rubus ulmifolius* Schott | [7] | Rosaceae | 4-Sep-2013 | Paúl de Madriz (40°07'47.4"N; 8°37'52.4"W) |
| *Smilax azorica* H. Schaef. & P. Schoenfelder | Endemic | *Smilax aspera* L. | [8, 9] | Smilacaceae | 9-Sep-2013 | Larçã (40°18'46.7''N; 8°24'14.5''W) |
| *Taxus baccata* L. | Non-endemic | *Taxus baccata* L. | - | Taxaceae | 6-Nov-2013 | Seia (40°25'07.5''N; 7°42'08.9''W) |
| *Vaccinium cylindraceum* Sm. | Endemic | *Vaccinium cylindraceum* Sm. *(fruits of V. myrtillus could not be found)* | - | Ericaceae | 10-Oct-2013 | Nordeste, Azores (37°49'45.1"N; 25°09'04.1"W) |
| *Viburnum treleasei* Gand. | Endemic | *Viburnum tinus* L. | [10] | Adoxaceae | 2-Nov-2013 | Benfeita (40°13'42.0''N; 7°56'23.1''W) |

**References**

1. Alonso C, Herrera CM. Back-and-forth hermaphroditism: phylogenetic context of reproductive system evolution in subdioecious *Daphne laureola*. Evolution. 2011;65(6):1680-92. doi: 10.1111/j.1558-5646.2011.01246.x.

2. Valcárcel V, Fiz O, Vargas P. Chloroplast and nuclear evidence for multiple origins of polyploids and diploids of *Hedera* (Araliaceae) in the Mediterranean basin. Mol Phylogenet Evol. 2003;27(1):1-20.

3. Selbach-Schnadelbach A, Cavalli SS, Manen J-F, Coelho GC, De Souza-Chies TT. New information for Ilex phylogenetics based on the plastid psbA-trnH intergenic spacer (Aquifoliaceae). Bot J Linn Soc. 2009;159(1):182-93. doi: 10.1111/j.1095-8339.2008.00898.x.

4. Rumeu B, Caujapé-Castells J, Blanco-Pastor JL, Jaén-Molina R, Nogales M, Elias RB, et al. The colonization history of *Juniperus brevifolia* (Cupressaceae) in the Azores Islands. PLoS ONE. 2011;6(11):e27697. doi: 10.1371/journal.pone.0027697.

5. Rodríguez-Sánchez F, Guzmán B, Valido A, Vargas P, Arroyo J. Late Neogene history of the laurel tree (*Laurus* L., Lauraceae) based on phylogeographical analyses of Mediterranean and Macaronesian populations. Journal of Biogeography. 2009;36:1270-81.

6. García-Verdugo C, Calleja JA, Vargas P, Silva L, Moreira O, Pulido F. Polyploidy and microsatellite variation in the relict tree Prunus lusitanica L.: how effective are refugia in preserving genotypic diversity of clonal taxa? Mol Ecol. 2013;22(6):1546-57. doi: 10.1111/mec.12194.

7. Lopes MS, Maciel GB, Mendonça D, Gil FS, da Câmara Machado A. Isolation and characterization of simple sequence repeat loci in Rubus hochstetterorum and their use in other species from the Rosaceae family. Mol Ecol Notes. 2006;6(3):750-2. doi: 10.1111/j.1471-8286.2006.01329.x.

8. Schaefer H, Schoenfelder P. *Smilax canariensis*, *S. azorica* (Smilacaceae) and the genus *Smilax* in Europe. In: Beltrán Tejera E, Afonso-Carrillo J, Gallo AG, Delgado OR, editors. Homenaje al Profesor Dr Wolfredo Wildpret de la Torre: Instituto de Estudios Canarios; 2009. p. 297-307.

9. Qi Z, Cameron KM, Li P, Zhao Y, Chen S, Chen G, et al. Phylogenetics, character evolution, and distribution patterns of the greenbriers, Smilacaceae (Liliales), a near-cosmopolitan family of monocots. Bot J Linn Soc. 2013;173(4):535-48. doi: 10.1111/boj.12096.

10. Moura M, Silva L, Caujapé-Castells J. Population genetics in the conservation of the Azorean shrub *Viburnum treleasei* Gand. Plant Syst Evol. 2013;299(10):1809-17. doi: 10.1007/s00606-013-0836-4.
