# Supplementary material for: On the Limited Potential of Azorean Fleshy Fruits for Oceanic Dispersal
Source: PLoS One. 2015 Oct 14;10(10):e0138882. doi: 10.1371/journal.pone.0138882 (PMC4605496; doi:10.1371/journal.pone.0138882)
Supplement: S2 Table — (DOCX) [file pone.0138882.s005.docx]

**S2 Table**

| **Min.** | **Average** | **Max.** | **References** |
| --- | --- | --- | --- |
| 0.3 | 1.6 | 2.9 | [1] |
| 10 | 20 | 30 | [2]^a^ |
| 2.2 | 3.3 | 4.4 | [3] |
| - | 3 | - | [4] |
| - | 3.3 | - | [5] |

^a^ General ocean current

**References**

1. Martins SM, Hamann M, Fiúza AFG. Surface circulation in the eastern North Atlantic, from drifters and altimetry. J Geophys R. 2002; 107(C12): 10-1-10-22
2. Nathan R, Schurr FM, Spiegel O, Steinitz O, Trakhtenbrot A, Tsoar A. Mechanisms of long-distance seed dispersal. Trends Ecol Evol. 2008; 23(11):638-47. doi: <http://dx.doi.org/10.1016/j.tree.2008.08.003>
3. Zhou M, Paduan JD, Niiler PP. Surface currents in the Canary Basin from drifter observations. J Geophys R. 2000; 105(C9): 21893-21911
4. Álvarez-Salgado XA, Figueiras FG, Pérez FF, Groom S, Nogueira E, Borges AV, et al. The Portugal coastal counter current off NW Spain: new insights on its biogeochemical variability. Prog Oceanogr. 2003; 56(2): 281-321
5. Seidov D, Sarnthein M, Stattegger K, Prien R, Weinelt M. North Atlantic ocean circulation during the last glacial maximum and subsequent meltwater event: A numerical model. J Geophys R. 1996; 101(C7): 16305-16332
