# Supplementary material for: On the Limited Potential of Azorean Fleshy Fruits for Oceanic Dispersal
Source: PLoS One. 2015 Oct 14;10(10):e0138882. doi: 10.1371/journal.pone.0138882 (PMC4605496; doi:10.1371/journal.pone.0138882)
Supplement: S3 Table — Taxonomy of Azorean species followed [34], European taxonomy followed the Flora Europaea of selected plant families [43]. The proportion of viable and germinated seeds between the two treatments and the control have been tested with G-tests (d.f. = 1, α = 0.05) and statistically significant differences highlighted in bold. (DOCX) [file pone.0138882.s006.docx]

**S3 Table**

|  | **Viability % (n of viable/n tested seeds)** | | | | | **Germination % (n of germinations/n seeds planted)** | | | | |
| --- | --- | --- | --- | --- | --- | --- | --- | --- | --- | --- |
| **Plant Species** | **Control (no immersion)** | **15 days** | | **60 days** | | **Control (no immersion)** | **15 days** | | **60 days** | |
|  |  | **Sank** | **Floating** | **Sank** | **Floating** |  | **Sank** | **Floating** | **Sank** | **Floating** |
| *Corema album* | 55.0 (11/20) |  | 70.6 (12/17)  G=0.22, p=0.639 | 15.4 (2/13)  G=2.67, p=0.102 | 100.0 (5/5)  G=0.66, p=0.417 | 0 (0/30) | 0 (0/30) |  | 0 (0/30) |  |
| *Daphne gnidium* | 76.5 (13/17) | 0 (0/19) |  | 0 (0/20) |  | 62.0 (18/29) | 0 (0/30) |  | 0 (0/30) |  |
| *Frangula alnus* | 100.0 (20/20) | 75.0 (18/24)  G=0.42, p=0.517 |  | 71.4 (15/21)  G=0.53, p=0.467 |  | 30.0 (9/30) | 70.0 (21/30)  G=3.33, p=0.068 |  | 86.7 (26/30)  **G=5.56, p=0.018** |  |
| *Hedera hibernica* | 91.7 (22/24) | 0 (0/24) |  | 0 (0/24) |  | 90.0 (27/30) | 0 (0/30) |  | 0 (0/30) |  |
| *Ilex aquifolium* | 75.0 (15/20) | 89.5 (17/19)  G=0.14, p=0.712 |  | 5.0 (1/20)  **G=11.16, p<0.001** |  | 0 (0/30) | 0 (0/30) |  | 0 (0/30) |  |
| *Juniperus oxycedrus* | 64.7 (11/17) | 0 (0/4) | 100.0 (1/1)  G=0.09, p=0.767 | 18.2 (2/11)  G=2.54, p=0.111 | 0 (0/1) | 0 (0/30) | 0 (0/8) | 0 (0/24) | 0 (0/30) |  |
| *Laurus nobilis* | 100.0 (24/24) | 90.0 (18/20)  G=0.06, p=0.808 |  | 85.0 (17/20)  G=0.14, p=0.711 |  | 100.0 (30/30) | 60.0 (18/30)  G=1.69, p=0.193 |  | 0 (0/30) |  |
| *Morella faya* | 100.0 (18/18) | 94.4 (17/18)  G=0.01, p=0.904 |  | 13.3 (2/15)  **G=8.03, p=0.004** |  | 26.7 (8/30) | 83.3 (25/30)  **G=6.07, p=0.014** |  | 0 (0/30) |  |
| *Prunus lusitanica* | 100.0 (24/24) | 35.0 (7/20)  **G=4.26, p=0.039** |  | 0 (0/24) |  | 40.0 (12/30) | 0 (0/30) |  | 0 (0/30) |  |
| *Rubus ulmifolius* | 94.4 (17/18) | 57.9 (11/19)  G=0.94, p=0.333 |  | 40.9 (9/22)  G=2.66, p=0.103 |  | 23.3 (7/30) | 30.0 (9/30)  G=0.20, p=0.656 |  | 23.3 (7/30) |  |
| *Smilax aspera* | 31.6 (6/19) | 57.9 (11/19)  G=1.04, p=0.308 |  | 8.3 (2/24)  G=2.66, p=0.103 |  | 13.3 (4/30) | 20.0 (6/30)  G=0.35, p=0.557 |  | 0 (0/30) |  |
| *Taxus baccata* | 85.7 (18/21) | 77.8 (14/18)  G=0.41, p=0.839 |  | 23.5 (4/17)  **G=4.57, p=0.032** |  | 0 (0/30) | 0 (0/29) | 0 (0/1) | 0 (0/30) |  |
| *Vaccinium cylindraceum* | 77.3 (17/22) | 13.0 (3/23)  **G=8.22, p=0.004** |  | 0 (0/24) |  | 0 (0/30) | 0 (0/30) |  | 0 (0/30) |  |
| *Viburnum tinus* | 84.2 (16/19) | 66.7(12/18)  G=0.26, p=0643 |  | 28.6 (4/15)  G=3.37, p=0.066 |  | 3.3 (1/30) | 0 (0/30) |  | 0 (0/30) |  |
